# Venetoclax Plus Intensified Chemoimmunotherapy as a Bridge to Allogeneic Stem Cell Transplantation in Richter Syndrome: Report of Two Cases

**DOI:** 10.3390/hematolrep16040075

**Published:** 2024-12-13

**Authors:** Enrico Derenzini, Alessandro Cignetti, Valentina Tabanelli, Daniela Gottardi, Elvira Gerbino, Anna Vanazzi, Simona Sammassimo, Alessio Maria Edoardo Maraglino, Federica Melle, Giovanna Motta, Daniela Malengo, Emanuela Omodeo Salè, Lisa Bonello, Rocco Pastano, Stefano Pileri, Fabrizio Carnevale Schianca, Corrado Tarella

**Affiliations:** 1 Oncohematology Division, IEO European Institute of Oncology IRCCS, Via Ripamonti 435, 20141 Milan, Italy; anna.vanazzi@ieo.it (A.V.); simona.sammassimo@ieo.it (S.S.); alessiomariaedoardomaraglino@ieo.it (A.M.E.M.); rocco.pastano@ieo.it (R.P.); corrado.tarella@ieo.it (C.T.); 2 Department of Health Sciences, University of Milan, Via Antonio di Rudinì 8, 20142 Milan, Italy; 3 Hematology and Cell Therapy, A.O. Mauriziano-Umberto I Hospital, Largo Filippo Turati 62, 10128 Turin, Italy; alcignet@gmail.com (A.C.); dgottardi@mauriziano.it (D.G.); 4 Division of Haematopathology, IEO European Institute of Oncology IRCCS, Via Ripamonti 435, 20141 Milan, Italy; valentina.tabanelli@ieo.it (V.T.); federica.melle@ieo.it (F.M.); mottagiovanna1@gmail.com (G.M.); stefano.pileri@ieo.it (S.P.); 5 Department of Experimental Oncology, IEO European Institute of Oncology IRCCS, Via Ripamonti 435, 20141 Milan, Italy; elvira.gerbino@ieo.it; 6 Pharmacy, IEO European Institute of Oncology IRCCS, Via Ripamonti 435, 20141 Milan, Italy; daniela.malengo@ieo.it (D.M.); eomodeo@ieo.it (E.O.S.); 7 Department of Molecular Biotechnology and Health Sciences, University of Turin, Via Nizza 52, 10126 Turin, Italy; lisa.bonello@unito.it; 8 Turin Metropolitan Transplant Center, Hematopoietic Stem Cells Unit, Department of Medical Oncology, Candiolo Cancer Institute, FPO-IRCCS, SP142, Km 3.95, 10060 Candiolo, Italy; fabrizio.carnevale@ircc.it

**Keywords:** Richter syndrome, chronic lymphoid leukemia, venetoclax, chemotherapy, GMALL regimen

## Abstract

**Background:** Richter syndrome (RS) represents a major unmet need in the lymphoma field, being refractory to chemoimmunotherapy and targeted agents. The BCL-2 inhibitor venetoclax in combination with dose-adjusted EPOCH-R chemoimmunotherapy showed promising efficacy in patients affected by RS. However, responses were not durable, suggesting the need for further treatment optimization. **Methods:** Here, we report two cases of RS achieving long-term complete remission with intensified chemoimmunotherapy (Rituximab-G-MALL B-ALL/NHL2002 regimen) plus venetoclax induction, followed by haploidentical hematopoietic stem cell transplant (allo-HSCT). Venetoclax was given continuously for 14 consecutive days after every Rituximab-G-MALL cycle in off-label use. An accelerated venetoclax rump-up schedule was used in both patients to reach the maximal dose. Maximal venetoclax dose was 300 mg and 400 mg in patient 1 and patient 2, respectively. **Results:** The combined treatment was well tolerated, with no major infective complications or non-hematological toxicities. In both patients, immunosuppression was discontinued within day 180 after transplant with no graft-versus-host-disease flares. Both patients are alive and in continuous complete remission after 60 and 72 months following allo-HSCT. **Conclusions:** This report supports the feasibility of a combination treatment with BCL-2 inhibitors and intensive chemoimmunotherapy as a bridge to allo-HSCT in RS.

## 1. Introduction

Richter syndrome (RS) is a very aggressive disease, defined as the appearance of an aggressive lymphoma, in most cases a diffuse large B-cell lymphoma (DLBCL), in patients affected by chronic lymphocytic leukemia (CLL) [1,2]. RS represents a major unmet need in the lymphoma field, being refractory to standard and intensified chemoimmunotherapy approaches normally used for DLBCL and to novel CLL-targeted agents [Bruton tyrosine kinase (BTK) and BCL-2 inhibitors (BCL-2i) [3,4]. Although prior experiences with single-agent immune-checkpoint inhibitors did not confirm initial observations of promising activity [5,6], giving conflicting results in terms of efficacy, the combination of a venetoclax-based regimen and atezolizumab recently showed proof of activity in RS, with complete responses (CRs) observed in one-third of patients [7].

Allogeneic hematopoietic stem cell transplantation (allo-HSCT) can lead to long-term remissions in the minority of patients responding to induction therapy [8]. However, the lack of effective induction regimens represents a major challenge in RS therapy: standard chemoimmunotherapy from the one hand [9] and single-agent BTKi or BCL-2i from the other [10,11] have shown very limited efficacy. Recently, combinatory approaches of venetoclax plus chemoimmunotherapy showed promising results in terms of CR rates: a phase 2 trial of dose-adjusted (DA)-EPOCH-R (etoposide, prednisolone, vincristine, doxorubicin, cyclophosphamide, Rituximab) plus venetoclax resulted in a 50% CR rate, which, however, translated in a 25% two-year progression-free survival (PFS) rate [12]. Although CR data compare favorably with historical case series, the poor durability of response highlights the need for further treatment optimization. Here, we report two cases of RS in long-term complete remission following haploidentical allo-HSCT performed after venetoclax plus intensified chemoimmunotherapy [R-G-MALL (German Multicenter Study Group for Adult ALL) B-ALL/NHL 2002 regimen] [13] induction treatment.

## 2. Materials and Methods

### 2.1. Patients

The two patients included in this study were treated at the European Institute of Oncology in Milan (Case 1) and at Mauriziano Hospital in Turin with the allo-HSCT procedure performed at Candiolo Cancer Institute (Case 2). Both patients received off-label venetoclax in combination with R-GMALL regimen (Rituximab, Vincristine, Ifosphamide, Methotrexate, Cytarabine, Etoposide, Doxorubicin) [13]. This study was approved by the Institutional review Boards of the participating centers (approval no. R633/17-IEO669, Milan, Italy) and carried out in accordance with the principles of the Helsinki declaration. Off-label use of venetoclax was approved by the Institutional review board. Patients signed written informed consent.

### 2.2. Clonal Relationship, IGHV, and TP53 Mutational Status Analyses

To establish the clonal relationship between the CLL and the RS components, we performed a sequencing analysis of the immunoglobulin-heavy-chain Ig VDJ genes.

Genomic DNA was extracted from peripheral blood (CLL samples) and formalin-fixed paraffin-embedded (FFPE) lymph node biopsies (RS samples) using MagCore^®^ Automated Extraction Instruments (MagCore^®^ Genomic DNA FFPE One-Step Kit and MagCore^®^ Genomic DNA Whole Blood Kit, RBC Bioscience Corp. New Taipei City, Taiwan). The instrument also measures the yield and purity of the extracted DNA.

Clonality assays were conducted following the BIOMED-2 protocol and evaluated using GeneMapper^®^ Software version 5.0 (Thermo Fisher Scientific, Waltham, MA, USA). A PCR-based assay for *IG* V(D)J rearrangements was performed with specific primers beginning with the leader and the framework-region 1 (FR1) zones [14,15]. These primers were used in conjunction with a single JH consensus primer. Qualified PCR products were subjected to direct sequencing on both strands to assess IGHV mutational status using the IGH Somatic Hypermutation Assay version 2.0 (Invivoscribe, Inc., San Diego, CA, USA), according to the manufacturer’s protocol. CLL and RS sequences were analyzed using the IMTG databases and the IMTG/V-QUEST tool (http://www.imtg.org accessed on 18 May 2017 and 16 May 2018; IMGT/V-QUEST program version: 3.5.16; IMGT/V-QUEST reference directory release: 202005-3). IGHV sequences were considered mutated if the homology to the corresponding germline gene was <98% [16].

*TP53* mutational status was assessed with Sanger sequencing according to the European Research Initiative on Chronic Lymphocytic Leukemia (ERIC) guidelines [17] using a 3500xl DX Genetic Analyzer (Applied Biosystems-Thermo Fisher). Electropherograms were visually inspected using the web-based tool, GLASS (http://bat.infspire.org/genomepd/glass/, accessed on 18 May 2017 and 16 May 2018), and detected variants were subsequently verified using an IARC TP53 database (http://p53.iarc.fr/TP53GeneVariations.aspx, accessed on 18 May 2017 and 16 May 2018).

### 2.3. Immunohistochemistry

After deparaffinization and antigen retrieval (PTLink at 92 °C for 5 min in EnVision Flex Target Retrieval Solution High pH), immunohistochemistry was performed on 2 μm-thick tissue sections from the FFPE blocks using the on the Dako AutoStainer Link48 (Dako Agilent, Glostrup, Denmark). The slides were then incubated with hematoxylin and Fast Red^®^ chromogen. The source, clone, and dilution of the antibodies are detailed in the respective figure legends.

## 3. Results

### 3.1. Case 1

A 58-year-old man was diagnosed with RS in May 2018 after an initial diagnosis of 17p deleted, with a TP53 mutant and an IGHV-unmutated CLL. At CLL onset, there was a mild lymphocytosis (13,930 lymphocytes/μL), and a CT scan demonstrated multiple enlarged abdominal adenopathies reaching a 6 cm diameter. A lymph node biopsy was consistent with a diagnosis of small lymphocytic B-cell lymphoma (SLL). The RAI stage at diagnosis was 1 (Binet B). His past medical history was notable for ulcerative colitis (treated with 5-aminosalicylate since the age of 51), a transient ischemic attack at the age of 43, a restless leg syndrome, and arterial hypertension requiring therapy with valsartan.

**Figure 1:**
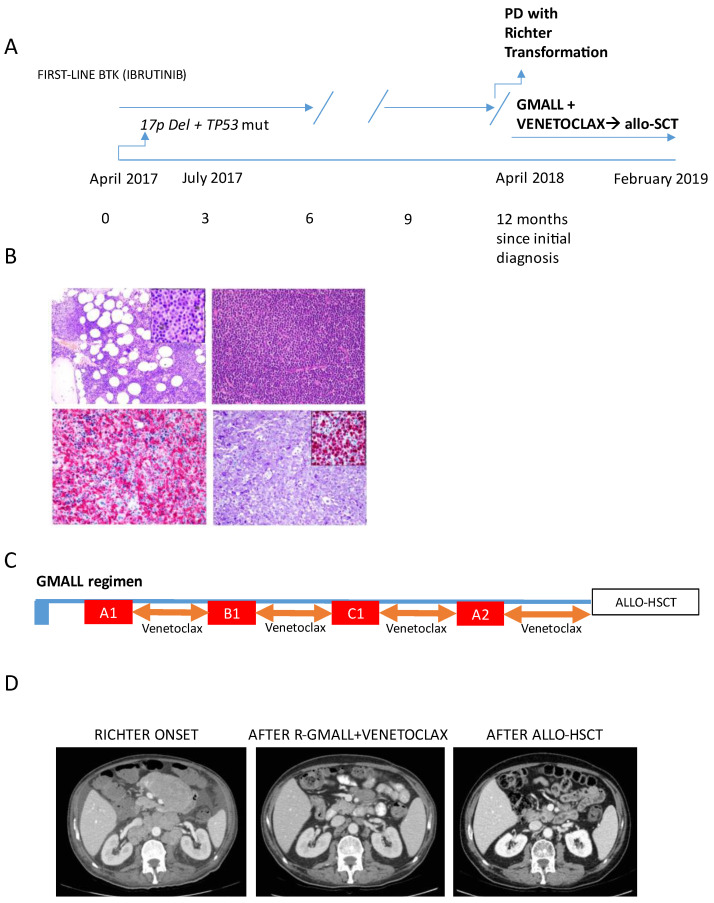
Main clinical and histological characteristics of Case 1. (**A**) Medical history and treatments received from initial diagnosis to Richter Transformation and allo-SCT. (**B**) (upper left panel) CLL, bone marrow biopsy with interstitial and nodular infiltrate of small-sized leukemic cells (Giemsa stain; original magnification 100×). Inset: high-power magnification of a pale proliferation center, composed by larger prolymphocyte-like cells (Giemsa stain; original magnification 600×). (upper right panel) CLL/SLL, lymph node involvement: pseudo-follicular growth pattern with pale proliferation centers (H&E stain, original magnification, 200×). (lower left panel) Bone marrow biopsy, hemophagocytic syndrome: CD163 stain (Leica, 10D6, 1:100) highlights histiocytic hyperplasia and marked hemophagocytosis (original magnification 600×); (lower right panel) Richter syndrome: the lymph node structure is effaced by a diffuse proliferation of large lymphoid cells with atypical nuclei and prominent nucleoli (Giemsa stain; original magnification 600×), with high Ki-67 activity (inset; original magnification 600×. Ki-67: Dako, Mib-1, 1:100). (**C**) Treatment schedule: after an accelerated ramp-up, venetoclax was given at the daily dose of 300 mg starting from the next day after each chemotherapy course until the day before the next chemotherapy cycle; the patient received a total of 4 chemotherapy cycles (A1, B1, C1, A2 blocks). (**D**) Abdomen CT scans showing disease burden at RS onset and treatment response after the completion of venetoclax plus R-GMALL and after allo-HSCT (+6 months).

After a short wait-and-see period, the patient received first-line Ibrutinib. However, after one year, in May 2018, the patient complained of a nreappearance of B symptoms (fever, weight loss), associated with significant enlargement of cervical adenopathies (Figure 1A). The laboratory showed increased serum lactate dehydrogenase (LDH) levels. Suspecting histological transformation, a lymph node biopsy was performed, confirming the diagnosis of RS (Figure 1B), which was clonally related to the original CLL, as determined by an analysis of the immunoglobulin heavy chain Ig VDJ genes. A bone marrow biopsy showed CLL infiltration and evidence of a hemophagocytic syndrome. At this point, due to the rapid clinical deterioration, a combined treatment of venetoclax plus chemoimmunotherapy with the R-GMALL regimen was started. Venetoclax was given for 14 consecutive days, starting from the next day after each chemotherapy course until the day before the next chemotherapy cycle, with G-CSF support. An accelerated venetoclax ramp-up schedule (100 mg day 1, 200 mg day 2, 300 mg day 3) was used at first cycle (A1). (Figure 1C). After the first R-GMALL plus venetoclax course, the clinical conditions improved rapidly, with disappearance of fever and reduction in lymphadenopathies confirmed by an ecotomography scan. The patient was then treated with three additional courses of R-GMALL regimen (B1, C1, A2). There was a ten-day therapy delay at cycle 4 due to delayed hematologic recovery. A CT (represented in Figure 1D) and an FDG-PET scan performed after the fourth cycle showed a complete remission. Minimal residual disease (MRD) evaluation in the peripheral blood by flow cytometry was negative (<10^−4^). Given the achievement of complete remission, the patient underwent allo-HSCT with a haplo-identical related donor, according to the original Baltimore schedule [18] (Cy 14.5 mg/kg/day i.v. on days −6 and −5, fludarabine 30 mg/m^2^/day i.v. on days −6 to −2, and 200 cGy of TBI on day −1 with 50 mg/kg Cy administered on days +3 and +4). The transplant procedure was well tolerated, with no major complications. A CT scan performed 3 and 6 months after allo-HSCT demonstrated further reduction in residual abdominal masses (Figure 1D). After 60 months from allo-HSCT, the patient is alive and in continuous complete remission.

### 3.2. Case 2

**Figure 2:**
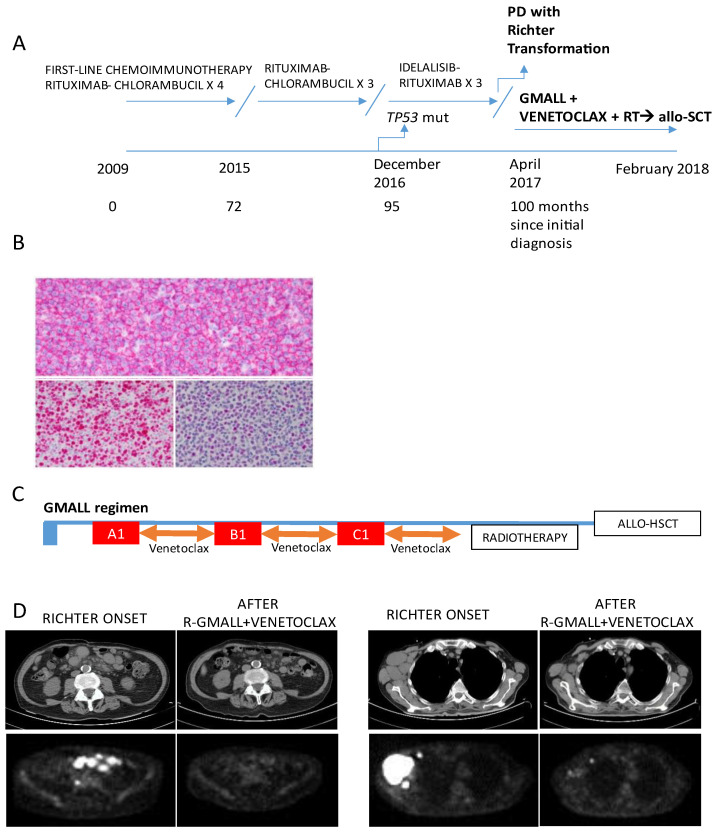
Main clinical and histological characteristics of Case 2: (**A**) Medical history and treatments received from initial diagnosis to Richter transformation and allo-SCT. (**B**) Richter syndrome: (upper panel) the lymph node structure is effaced by a diffuse proliferation of CD20+ large-sized lymphocytes with pleomorphic nuclei and prominent nucleoli (CD20: Dako, L26, 1:300), with high Ki-67 activity (depicted in (lower left panel) Ki-67: Dako, Mib-1, 1:100), and variable expression of p53 (represented in (lower right panel)) (original magnification, 400× and 200×. p53: Leica, DO7, 1:150). (**C**) Treatment schedule: after an accelerated ramp-up, venetoclax was given at the daily dose of 400 mg starting from the next day after each chemotherapy course until the day before the next chemotherapy cycle; the patient received a total of 3 chemotherapy cycles (A1, B1, C1 blocks). (**D**) CT and FDG-PET scans showing disease burden at RS onset and treatment response after the completion of venetoclax plus R-GMALL regimen.

A 74-year-old man was diagnosed with RS in 2017, 8 years after the initial diagnosis of a trisomy 12, *TP53* wild-type, CLL. IGHV status at diagnosis showed two clones with an unmutated and a mutated productive IGHV rearrangement respectively. Binet stage was C. His past medical history was notable for parossistic atrial fibrillation in chronic treatment with flecainid and prostatectomy for adenocarcinoma (in 2003). Prior CLL chemoimmunotherapy included first-line Rituximab–Chlorambucil in 2009 and Rituximab–Chlorambucil re-treatment in September 2015. In December 2016, multiple diffuse lymphadenopathies were documented with a total body CT scan, with abdominal lymph nodes up to 7 cm in diameter, associated with increased lymphocytosis and B symptoms. Molecular analysis of CLL revealed a *TP53* mutation. Patient was treated with Idelalisib–Rituximab, which was discontinued early, following the worsening of B symptoms (fever and weight loss) (Figure 2A). A FDG-PET/CT scan revealed multiple diffuse lymphadenopathies with an elevated standardized uptake value (SUV). An axillary nodal biopsy revealed transformation to Diffuse Large B cell lymphoma, thus configuring a diagnosis of RS (Figure 2B). Sequencing analysis of the immunoglobulin heavy chain Ig VDJ genes confirmed clonal relationship between the original CLL and RS. Two cycles of OxDHA (Oxaliplatin, dexamethasone, high dose ARA-C) were administered with no response, with persisting fever and further clinical deterioration. At this point, a combined treatment with venetoclax plus intensive chemo-immunotherapy (R-GMALL regimen) was started, with G-CSF support (Figure 2C). Again, venetoclax was given right after the completion of each first R-GMALL cycle. An accelerated ramp-up schedule was used at cycle A1 (100 mg day 1, 200 mg day 2, 400 mg day 3 and onward). An immediate reduction or disappearance of palpable lymphadenopathies was observed.

The patient was then treated with the B1 and C1 cycles of the GMALL regimen, and venetoclax was given again, starting from the next day after last chemotherapy infusion until the day before the next chemotherapy cycle. Bone marrow immunophenotyping performed after cycle 3 showed 0.2% CLL infiltration, and an FDG-PET scan showed a very good partial remission, with minimal residual FDG-PET uptake at two right axillary lymph nodes (Figure 2D).

Involved field radiotherapy on the residual FDG-PET positive nodal sites was administered, followed by haploidentical allo-HSCT consolidation, again according to the Baltimore schedule [18]. The transplant procedure was performed at Candiolo Cancer Institute with no major complications. A CT and an FDG-PET scan performed 4 and 7 months after allo-HSCT demonstrated a complete remission. At the last follow up, 72 months since allo-HSCT, the patient was alive and in continuous MRD-negative complete remission.

## 4. Discussion

Here, we report 2 cases of RS successfully treated with a combination of venetoclax plus intensive chemoimmunotherapy, (GMALL B-ALL/NHL 2002 regimen) [13], followed by haploidentical allo-HSCT. It is worth noting that both patients had a very high-risk RS, with several negative prognostic factors, such as clonal relationship between CLL and RS, high LDH, bulky masses, and *TP53* disruption [19].

The combination of GMALL plus venetoclax was very effective and well tolerated, and both patients were able to undergo allo-HSCT after 4 and 3 cycles of combined treatment, respectively. Given the lack of safety data regarding the co-administration of venetoclax and R-GMALL, venetoclax administration was started immediately after every GMALL cycle and given continuously for 14 days until the next GMALL cycle (from day 6 to day 20), with G-CSF support. This schedule has some biological justification, since exposure to chemotherapy could determine an apoptotic priming in RS cells by upregulating BH3 proteins [20], which could enhance venetoclax activity. Furthermore, the use of venetoclax as bridging therapy could be crucial in order to achieve optimal control of the underlying CLL before the allo-SCT procedure. Despite the addition of venetoclax, the R-GMALL regimen was well tolerated with no major infective complications or non-hematologic toxicities, and both patients underwent allo-HSCT in optimal clinical conditions. Both patients are presently alive and in CR at 60 and 72 months since allo-HSCT, respectively. In both patients, immunosuppression was discontinued within day 180 after transplant with no graft-versus-host-disease flares.

To the best of our knowledge, there are no reports on the activity of GMALL in RS, as this regimen has been developed and employed mainly in the setting of acute lymphoblastic leukemia and Burkitt lymphoma [13]. Therefore, it is difficult to draw conclusions on the added value of venetoclax in this particular setting. However, previous reports with regimens of similar intensity (hyperCVAD regimen) in RS gave disappointing results [9]. In this light, the present report supports the feasibility of adding BCL-2 inhibition in the setting of chemotherapy intensification, suggesting that BCL-2-inhibitors-based chemoimmunotherapy combinations could be an effective bridge to cellular therapies in RS, in line with available evidence from the literature [12,21]. Whether venetoclax should be preferentially combined with immunotherapy (such as checkpoints inhibitors) or with chemoimmunotherapy is still a matter of debate. The combination of venetoclax and chemoimmunotherapy may achieve 50% CR rates in RS [12,21], while the MOLTO study investigating the combination of venetoclax, obinutuzumab, and atezolizumab demonstrated CR in about one third of patients, with a fraction of them achieving long-term disease control [7]. The two cases described here and the available evidence could support the use of venetoclax plus chemoimmunotherapy as a bridging therapy in young RS patients when allo-SCT consolidation is planned, given the relatively high rates of complete remissions achieved with this strategy, enabling further transplant consolidation in a significant fraction of patients. On the contrary, venetoclax-based chemo-free combinations could be reserved to elderly patients, based on the lower toxicities and the observation that a fraction of patients could achieve long-term remission without further consolidation. Recently, T-cell redirection therapies with CAR-T cells [22] and bispecific antibodies [23,24] demonstrated promising efficacy in relapsed refractory RS, and clinical trials in first-line settings are underway. Whether the earlier use of T-cell redirection therapies in RS will result in higher rates of long-term CR will be determined in future studies.

## 5. Conclusions

The present report confirms the feasibility and long-term safety of combining venetoclax and intensive chemoimmunotherapy as an induction regimen and underscores the importance of cellular therapy consolidation after the achievement of CR in RS.

## Data Availability

All data generated or analyzed during this study are included in this published article.
